# Use of Procalcitonin to Guide Discontinuation of Antimicrobial Therapy in Patients with Persistent Intra-Abdominal Collections: A Case Series

**DOI:** 10.1155/2020/6342180

**Published:** 2020-01-09

**Authors:** Gabriel Motoa, Amy Pate, Carlos Franco-Paredes, Daniel B. Chastain, Andrés F. Henao-Martínez, Leila Hojat

**Affiliations:** ^1^Division of Infectious Diseases, Department of Medicine, University of Colorado Anschutz Medical Center, Aurora, CO, USA; ^2^Division of Preventive Medicine, Department of Family Medicine, University of Colorado Anschutz Medical Center, Aurora, CO, USA; ^3^Hospital Infantil de Mexico Federico Gomez, Mexico City, Mexico; ^4^Department of Clinical and Administrative Pharmacy, University of Georgia College of Pharmacy, Albany, GA, USA; ^5^Division of Infectious Diseases & HIV Medicine, University Hospitals Cleveland Medical Center, Case Western Reserve University, Cleveland, OH, USA

## Abstract

**Objective:**

Limited evidence exists for the use of procalcitonin (PCT) to guide the duration of antimicrobial therapy in patients with intra-abdominal abscesses (IAA). In this case series, we describe clinical presentations and outcomes using PCT to guide cessation of antimicrobial therapy in patients with persistent IAA who exhibited clinical improvement.

**Methods:**

A retrospective analysis of patients with IAA who had PCT levels available to review was performed in a tertiary academic teaching institution in the United States between 2017 and 2018. Demographics, clinical characteristics, and outcomes were obtained from the medical records. Patients were followed up for a minimum of 180 days after completion of antimicrobial therapy to determine if evidence of recurrence or mortality was present.

**Results:**

We identified four patients with IAA. They underwent early drainage of the source of infection and received empiric antimicrobial therapy according to individual risk factors and clinical scenarios. Antimicrobials were discontinued after clinical and radiographic improvement and evidence of normal PCT levels, regardless of the persistence of fluid collections. No evidence of recurrence or mortality was observed during the follow-up period.

**Conclusions:**

We found PCT to be a useful aid in the medical decision-making process to safely discontinue antimicrobial therapy in a series of patients with persistent intra-abdominal collections despite early drainage and appropriate course of antimicrobial therapy.

## 1. Introduction

Intra-abdominal infections are associated with high rates of complications and mortality [1, 2]. The general approach to patients with intra-abdominal infections includes opportune and adequate empirical antimicrobial therapy based on individual risk factors for resistant microorganisms in addition to identification and control of the infection source [3–6].

One of the most controversial topics among clinicians managing patients with intra-abdominal abscesses (IAA) is the appropriate duration of antimicrobial treatment once the source of infection has been controlled. It is known that prolonged usage of antimicrobials does not improve outcomes but instead is associated with the emergence of resistant pathogens, healthcare-associated infections, antimicrobial-related cost, and adverse effects [7, 8]. Given the aforementioned risks of prolonged antimicrobial therapy, current guidelines suggest shorter treatment duration and discontinuation of antimicrobials once inflammatory markers and other signs of infection resolve [9–11].

Procalcitonin (PCT) is currently one of the most commonly used biomarkers, and its role in guiding cessation of antimicrobial therapy in critically ill individuals with suspected infection in the intensive care unit has been extensively described [12, 13]. However, many of these studies excluded patients with intra-abdominal infections, and data from randomized trials regarding discontinuation of antimicrobials for IAA using PCT are lacking [14].

We describe the usage of PCT as a marker to guide cessation of antimicrobial therapy in patients with IAA who exhibited clinical improvement despite the presence of persistent fluid collections.

## 2. Methods

A retrospective analysis of hospital medical records, microbiology reports, and diagnostic imaging from a tertiary academic teaching institution in the United States between 2017 and 2018 was performed in four patients who had a diagnosis of IAA and PCT levels available to review.

Adults with intra-abdominal collections confirmed by abdominal computed tomography (CT) were included. Patients who underwent interventional drainage or surgery were considered eligible if they did not have a drain at admission. Exclusion criteria were as follows: age under 18 years, pancreatitis, intra-abdominal drains at admission, and absence of microbiological information after source control.

Demographics, clinical characteristics, and outcomes information were obtained from the medical records for patients who had PCT levels available to review. The methodology used to evaluate PCT was chemiluminescent microparticle immunoassay (CMIA)—B·R·A·H·M·S assay. A normal PCT value considered for this case series was ≤0.1 ng/mL, and levels ≥0.25 ng/mL were considered high. Patients were followed up for a minimum of 180 days after completion of antimicrobials to determine if evidence of clinical failure or mortality was present. Recurrence was defined as collections that increased in size despite a source control attempt and appropriate antimicrobial therapy, requiring a new drain placement or surgical intervention as per indication of the medical or surgical teams.

### 2.1. Case 1

**Figure 1:**
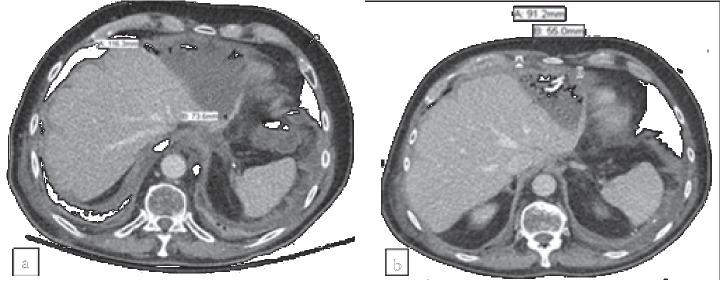
Computed tomography scan before starting antimicrobial therapy in Patient 1 shows a moderate volume of fluid and gas around the hepatic dome measuring approximately 11.6 × 7.4 cm (a). Computed tomography at time of antimicrobial completion shows a complex fluid collection containing a few air bubbles inseparable from the left lobe of liver with pigtail drain with interval decrease in size to 9.1 × 5.5 cm (b).

A 70-year-old male with a history of esophageal adenocarcinoma treated with chemotherapy and radiation was admitted with a complaint of severe abdominal pain and fever. The patient had undergone esophagectomy one month prior, which was complicated by conduit necrosis and empyema requiring thoracotomy. He had completed a 3-week course of broad-spectrum antimicrobials and had been discharged to a long-term acute care facility prior to his current presentation. Laboratory data at admission are shown in Table 1. Abdominal CT revealed a moderate volume of pneumoperitoneum and fluid around the left lobe of the liver as well as a rim-enhancing collection inferior to the left hepatic lobe with a focus of internal gas suspicious for an abscess (Figure 1(a)).

A CT-guided peritoneal drainage catheter was placed, and 150 mL of turbid yellow fluid was removed for diagnostic testing. Empiric antimicrobial therapy with vancomycin, piperacillin-tazobactam, and anidulafungin was initiated after the drainage. Blood cultures grew *Staphylococcus epidermidis* in two sets which cleared within 24 hours, while peritoneal fluid cultures and acid-fast bacilli smear of peritoneal fluid were negative. Vancomycin was continued upon discharge, and the other agents were switched to ertapenem and fluconazole. The patient exhibited clinical improvement and normal PCT levels at his two-week follow-up appointment, though repeat CT showed a persistent abscess mildly decreased in size compared to prior imaging (Figure 1(b)). Antimicrobials were discontinued at that time, and the abscess was completely resolved by day 79 when the drainage catheter was removed. No recurrence was noted at 180 days after the completion of treatment (Table 1).

### 2.2. Case 2

**Figure 2:**
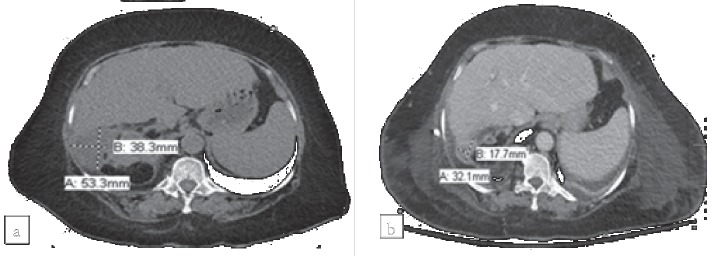
Computed tomography scan before starting antimicrobial therapy in Patient 2 demonstrates several central low-density fluid collections within the right perinephric space, below the liver margin, largest measuring approximately 5.3 × 3.8 cm (a). Computed tomography at time of antimicrobial completion shows a residual fluid collection measuring 3.2 × 1.8 cm (b).

A 63-year-old female with a history of nephrolithiasis and recently diagnosed diabetes mellitus presented with acute onset of fever and abdominal pain following a coughing episode. Exam was notable for a palpable right-sided abdominal mass and drainage from the umbilicus. Abdominal CT scan revealed a right retroperitoneal hematoma, xanthogranulomatous pyelonephritis, fluid collections within the superior right perinephric space, and rectus abdominis diastasis with a large bowel-containing hernia (Figure 2(a)).

The patient was started on vancomycin, cefepime, and metronidazole and underwent percutaneous drainage of the right flank hematoma with placement of drain. Subsequently, the right flank wound was surgically debrided, and a nephrostomy catheter was placed. The hematoma fluid aspirate cultures grew *Escherichia coli* and *Streptococcus anginosus*, intraoperative tissue cultures grew *E. coli*, and blood cultures remained negative. Antimicrobial therapy was initially narrowed to cefepime monotherapy but was later switched to ampicillin-sulbactam due to development of thrombocytopenia, and then to ceftriaxone for definitive inpatient treatment. Repeat CT scan three weeks after initial presentation showed a stable perinephric abscess with new small, peripheral collections in the right flank. The drain was exchanged, and a percutaneous nephrostomy was placed three days later. Urology considered a high surgical risk patient for nephrectomy at that moment and suggested that having the drains in place could be the best source control. The patient was discharged home with levofloxacin and metronidazole after 41 days of inpatient antimicrobial therapy. At four-week follow-up, CT showed a decrease in size of the right perinephric abscess as well as appropriate pigtail catheter placement, and levofloxacin was continued. One month after this visit, labs revealed a normal PCT, and CT showed a smaller, residual perinephric fluid collection (Figure 2(b)). Antimicrobials were discontinued after completing 91 days of therapy, with the drains remaining in place and producing clear fluid. The patient remained afebrile and asymptomatic during the following 180 days with plans for definitive treatment with nephrectomy.

### 2.3. Case 3

**Figure 3:**
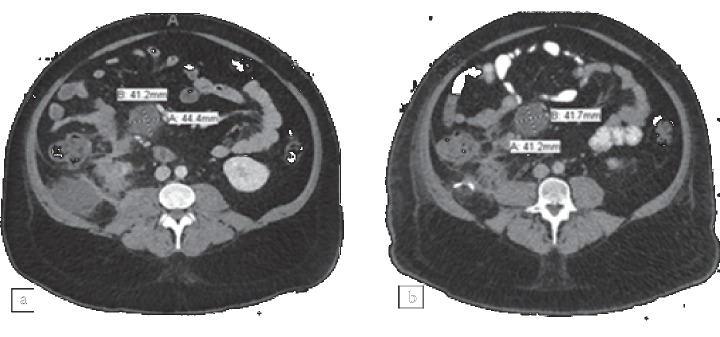
Computed tomography scan before starting antimicrobial therapy in Patient 3 shows a locule of complex fluid in the midline near the superior mesenteric vein measuring 4.4 × 4.1 cm (a). Computed tomography at time of antimicrobial completion shows a persistent phlegmonous collection measuring 4.2 × 4.1 cm (b).

A 31-year-old male with a history of diabetes mellitus presented to the emergency room with diffuse, stabbing abdominal pain, fever, and chills. On exam, the abdomen was tender in the right lower quadrant and periumbilical region with guarding and rebound tenderness. Abdominal CT showed a right psoas abscess with peritoneal extension into the right lower quadrant and an additional mass-like area of complex fluid in the middle abdomen (Figure 3(a)). The patient was started on empiric antimicrobial therapy with ceftriaxone, metronidazole, and vancomycin and underwent percutaneous drainage with drain placement in the right psoas abscess. Abscess cultures grew *Eikenella corrodens* and *Bacteroides fragilis*, and blood cultures were negative. Six days later, a new CT scan showed resolution of the retroperitoneal collection with no significant change in the right mesenteric collection, and the drain was removed. The patient was transitioned to ampicillin-sulbactam to be continued as an outpatient. Due to malfunction of the peripherally inserted central catheter, the patient was switched to amoxicillin-clavulanate after 34 days of parenteral therapy. At four-week follow-up, PCT was normal, and CT scan again showed no significant reduction in the size of the right mesenteric collection (Figure 3(b)). Antimicrobials were discontinued after a total of 54 days of therapy, and the patient showed clinical improvement with no recurrences at the 90- and 180-day follow-up appointments.

### 2.4. Case 4

**Figure 4:**
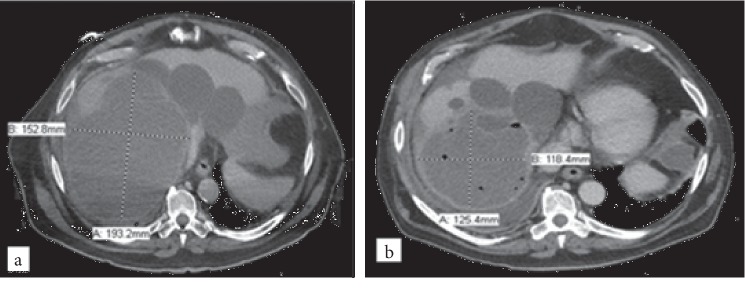
Computed tomography scan before starting antimicrobial therapy in Patient 4 demonstrates a dominant internally complex cyst in the right hepatic dome measuring 15.3 × 19.3 cm (a). Computed tomography at time of antimicrobial completion shows the cyst in the dome of liver with interval decrease in size after placement of drainage catheters, now measuring 11.8 × 12.5 cm (b).

A 74-year-old male with a history of polycystic liver and kidney disease and diabetes mellitus was admitted to an outside hospital with altered mental status and fever. He had undergone hepatic cyst drains placement and sclerosis one month prior, and those had been removed three weeks after their placement. Subsequently, he was admitted with sepsis and pneumonia and finally discharged on ampicillin-sulbactam three days prior to the current admission. Abdominal CT scan at the time of admission showed enlargement of a complex cyst located in the right hepatic dome compared to a study performed during the prior admission (Figure 4(a)). The patient was transferred to our hospital for definitive management. Due to the high perioperative risk, the cyst was aspirated and percutaneous drain was placed, with cultures later becoming positive for oxacillin-resistant *S. epidermidis* with susceptibility to vancomycin (MIC 1 *μ*g/mL). The patient was treated with daptomycin for three weeks, at which point the drain was exchanged and new cultures grew oxacillin-resistant *S. epidermidis* (MIC 2 *μ*g/mL to vancomycin). He was discharged on outpatient intravenous vancomycin, and CT scan three weeks later showed a persistent but smaller complex fluid collection. Fluid cultures obtained from the existing drain grew oxacillin-susceptible *S. epidermidis*, and the patient was transitioned to oral linezolid. Four weeks later, the patient was scheduled for an over-the-wire exchange and alcohol sclerosis of the liver cyst. At this time, CT scan showed a large, persistent fluid collection (Figure 4(b)), however, PCT was normal, and the patient was asymptomatic. Antimicrobials were discontinued after a total of ten weeks, and the patient had no recurrence of symptoms within 180 days of antimicrobial completion.

## 3. Discussion

We found PCT to be a useful aid in the medical decision-making process to safely discontinue antimicrobial therapy in a series of patients with persistent intra-abdominal collections despite early drainage and an appropriate course of antimicrobial therapy. Patients with IAA often receive unnecessarily prolonged antimicrobial therapy which has been associated with a higher risk of complications including extra-abdominal infections, selection for resistant pathogens, and mortality [15, 16]. However, many providers remain reluctant to discontinue antimicrobials in patients with persistent intra-abdominal fluid collections even in the setting of clinical improvement and downward trending or normal inflammatory markers [17, 18].

Preprocalcitonin is a 116-amino acid residue synthesized by thyroid C cells. Under normal circumstances, it is cleaved by endopeptidases into PCT, a 25-amino acid sequence. Posteriorly, the enzyme prohormone convertase catalyzes the formation of calcitonin, a 32-amino acid hormone involved in the regulation of calcium. The normal physiological serum concentration of PCT is less than 0.1 ng/mL. PCT levels ≥0.5 ng/mL are typically classified as elevated and suggestive of bacterial infection [19]. However, a cutoff of 0.25 ng/mL has been proposed as a better predictor of bacteremia in patients with community-acquired pneumonia or urinary tract infections [20, 21]. Production of PCT is activated in all parenchymal tissues when the levels of interleukin (IL)-1*β*, IL-6 and tumor necrosis factor (TNF)-alpha are elevated in response to bacterial infection and sepsis. On the other hand, some markers usually present in viral infections—as interferon-gamma—decrease levels of PCT, making PCT a good aid to differentiate bacterial from viral infections [22–24].

PCT levels peak by 6 to 22 hours and correlate with the severity of infection; as such, it has been described as a reliable biomarker for serial monitoring of bacterial infections and sepsis [25, 26]. Commonly, patients with septic shock and sepsis present with higher peak levels when compared with localized infections [19, 27]. After the resolution of infection, PCT levels usually drop by 50 percent every 24 to 36 hours. The PCT kinetics may differ significantly in patients with renal dysfunction; they commonly have higher baseline PCT levels and exhibit prolonged elimination rates [19, 28]. Additionally, PCT levels that increase over time or fail to normalize have been associated with a higher risk of mortality in patients with acute infectious diseases [29]. Other conditions may increase PCT levels as well including severe burns [30], trauma [31], cardiogenic shock [32], end-stage kidney disease [33], and surgery, particularly within the first two postoperative days [34].

Few studies have analyzed the use of PCT as a biomarker to guide antimicrobial therapy in IAA. Jeger et al. showed that PCT combined with abdominal CT scans might be able to differentiate uncomplicated diverticulitis from complicated diverticulitis involving abscess or perforation with high sensitivity and specificity, which suggests that PCT has the potential to identify the presence of IAA [35]. In this study, PCT values obtained on admission had only moderate sensitivity and specificity, requiring an additional level on day 2, which could be partially explained by the kinetics of PCT.

Observational studies have demonstrated that the use of PCT-based algorithms in the setting of intra-abdominal infection reduces the duration of antimicrobial exposure without increasing infectious complications. Chomik et al. found a high negative predictive value for systemic infections in patients with elevated PCT levels after elective colorectal surgeries. Additionally, they observed a significant reduction in the frequency of postoperative infections and the duration of antimicrobial treatment when a preemptive antibiotic treatment was administered [36]. Similarly, Huang et al. described a reduction of 3 days in the duration of antimicrobial treatment when a PCT algorithm was used in patients with secondary peritonitis [37].

Most recently, the usage of PCT was reported to lead to a reduction of 2.6 days in the duration of antimicrobial therapy in patients with peritonitis requiring surgical management, with no increased risk of recurrent infections [38]. Data from a recent meta-analysis also indicated that PCT-guided antimicrobial treatment in the intensive care unit decreased mortality, including the subgroup of individuals with intra-abdominal infection, although this subgroup did not have a decrease in the length of antimicrobial duration compared to other sites of infection [39].

Conversely, a randomized controlled study that evaluated PCT levels to guide postoperative antimicrobial therapy in patients with peritonitis did not find significant differences in the duration of antimicrobials in the group with PCT guidance compared to the group that received standard treatment [40]. However, this study included a broad range of underlying etiologies and was not powered for subgroup analysis which could explain the lack of statistical significance in the group with peritonitis secondary to gastrointestinal perforation.

**Figure 5:**
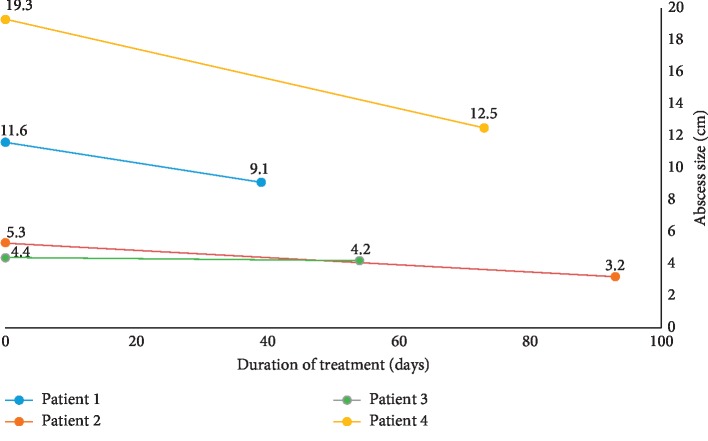
Source control attempt and antimicrobial therapy in patients with persistent intra-abdominal collections.

This case series represents the first report of PCT usage to guide therapy in patients with IAA. We identified four patients with IAA; three patients had diabetes mellitus, and two out of the four patients had recently been hospitalized and treated with antimicrobials. The cases differed in that they had a wide age range (median 66.5 years), varied suspected sources of infection, and significant microbiological diversity. All cases were initially treated based on current management recommendations with broad-spectrum antimicrobial coverage and early drainage of the suspected source of infection [41]. The median duration of antimicrobial therapy the patients received before PCT was 63.5 days (interquartile range, 50–78 days). Despite this management, all patients had sizeable persistent fluid collections, up to 12 cm in Case 4. Antimicrobials were discontinued regardless of these persistent collections, and all patients exhibited a favorable outcome with survival at 180 days with no evidence of recurrence of symptoms. Earlier antimicrobial cessation would likely have been possible, but the optimal duration of antimicrobial therapy in these cases remains uncertain (Figure 5).

This study adds to the growing body of literature regarding PCT-guided antimicrobial treatment in that it focuses on patients with IAA as a complication of intra-abdominal or systemic infection. This represents a particularly complicated subgroup of individuals who have not been analyzed specifically in clinical trials and are often excluded from critical care studies. Moreover, this population is often treated for extended durations, up to several months in some cases, which differs from populations typically included in trials of PCT-guided treatment. Thus, the potential for reduction in costs, adverse effects, and the emergence of antimicrobial resistance is much greater in patients with IAA. An additional strength of this case series is that the follow-up period extended to at least six months in all patients and up to one year in the majority, which renders any missed complication of a shortened antimicrobial course unlikely.

Limitations of this study include that PCT was not checked more frequently throughout the treatment course. However, a normal PCT level complements other clinical factors which can be taken into consideration at any point during the treatment course, giving the provider an additional tool to assist with clinical decision-making. Also, treating physicians saw PCT as a real-time point-of-care tool to aid in the medical decision process, rather than a conventional inflammatory trending marker. Further, this is somewhat reflective of clinical practice in that laboratory values are often not regularly monitored after hospital discharge, particularly in the case of inflammatory markers. Finally, without a control group, the findings from this report need to be interpreted with caution.

Future studies involving PCT-guided antimicrobial treatment should focus on patients with IAA and other complicated infections typically treated for extended durations, as this subset has the potential for a substantial benefit from shortened antimicrobial courses.

## 4. Conclusions

In our series of patients with IAA and persistent fluid collections, discontinuing antimicrobial therapy did not result in negative clinical outcomes after early source control was attempted and PCT levels had normalized. Further investigation is necessary to establish the definitive role of PCT as a tool to guide the duration of antimicrobial therapy in these patients.

## Figures and Tables

**Table 1:** Demographic characteristics, clinical manifestations, and outcomes of patients with intra-abdominal abscess.

	Patient 1	Patient 2	Patient 3	Patient 4
Age	70	63	31	74
Sex	Male	Female	Male	Male
Race	White	Black	Black	White
Admission labs				
WBC, 1000 per mm^3^	9.6	14.5	14.4	9.4
Platelets, 1000 per mm^3^	360	321	359	285
Lactate, mmol/L	2.3	5.9	5.7	2.5
Isolated bacteria	*Staphylococcus epidermidis* (blood)	*Escherichia coli*, *Streptococcus anginosus*	*Eikenella corrodens*, *Bacteroides fragilis*	*Staphylococcus epidermidis*
Suspected site of origin	Small bowel	Perinephric abscess	Colon vs appendix	Liver and kidney cysts
Procedure to control the source of infection	Percutaneous drainage	Percutaneous drainage	Percutaneous drainage	Percutaneous drainage
Abscess size (cm) at diagnosis	11.6 × 7.4	5.3 × 3.8	5.7 × 6.2 (psoas), 4.1 × 4.4 (mesenteric)	19.3 × 15.3
Abscess size (cm) when antimicrobials were stopped	9.1 × 5.5	3.2 × 1.8	4.1 × 4.2 (mesenteric)	11.8 × 12.5
Procalcitonin levels (ng/mL) when antimicrobials were stopped	0.07	0.05	0.03	0.04
Total duration of antimicrobial therapy (days)	39	93	54	73
Length of hospital stay (days)	7	41	7	12
Readmission at 30 days	No	No	No	No
Mortality				
30-day	No	No	No	No
90-day	No	No	No	No
180-day	No	No	No	No
1-year	No	No	No	N/A

^a^The total duration of antimicrobial therapy was defined as the sum of calendar days of inpatient plus postdischarge antimicrobial administration. WBC, white blood cells.
